# Navigating Disease Management: A Comprehensive Review of the De Ritis Ratio in Clinical Medicine

**DOI:** 10.7759/cureus.64447

**Published:** 2024-07-13

**Authors:** Suhail M Shaikh, Anuj Varma, Sunil Kumar, Sourya Acharya, Rajvardhan Patil

**Affiliations:** 1 Internal Medicine, Jawaharlal Nehru Medical College, Datta Meghe Institute of Higher Education and Research, Wardha, IND

**Keywords:** clinical medicine, prognostic indicator, diagnostic biomarker, liver diseases, disease management, de ritis ratio

## Abstract

The De Ritis ratio, defined as the serum aspartate aminotransferase (AST) to alanine aminotransferase (ALT) ratio, is a widely recognized biochemical marker with significant applications in diagnosing and managing various diseases, particularly liver disorders. This comprehensive review synthesizes current knowledge surrounding the clinical relevance of the De Ritis ratio, examining its historical development, diagnostic utility, and prognostic significance across various medical conditions, including liver diseases, cardiovascular disorders, and muscular pathologies. Through an in-depth analysis of literature spanning several decades, this review highlights the role of the De Ritis ratio not only in differential diagnosis but also as a prognostic indicator for disease progression and patient outcomes. The ratio's ability to distinguish between different types of liver pathology, aid in early disease detection, and its potential use in monitoring treatment response are discussed. Additionally, the review addresses the methodological considerations, such as confounding factors and interpretation challenges, that impact the clinical utility of the De Ritis ratio. Given the evolving landscape of clinical diagnostics and the push toward more personalized medicine, the review concludes with recommendations for further research. These include longitudinal studies to explore the ratio's changes over time, comparative research across diverse populations, and technological integration to enhance diagnostic accuracy and patient care. This review aims to reaffirm the importance of the De Ritis ratio in modern clinical practice and encourages continued exploration into its potential applications and benefits in healthcare.

## Introduction and background

Disease management encompasses a broad spectrum of strategies to prevent, diagnose, treat, and monitor various medical conditions. It involves a multidisciplinary approach that integrates medical interventions, lifestyle modifications, and patient education to optimize health outcomes and quality of life [1]. Biomarkers play a crucial role in clinical medicine by providing objective indicators of biological processes, disease progression, and treatment response. These measurable characteristics can range from molecules in bodily fluids to imaging findings and genetic markers. Biomarkers are valuable tools for disease diagnosis, prognosis, and monitoring, facilitating personalized treatment approaches and improving patient care [2].

The De Ritis ratio, also known as the AST/ALT ratio, is a simple biomarker derived from the levels of two liver enzymes: aspartate aminotransferase (AST) and alanine aminotransferase (ALT). Initially proposed by Fernando De Ritis in 1957, this ratio has gained prominence in assessing liver function and evaluating various hepatobiliary disorders [3]. This comprehensive review aims to explore the De Ritis ratio's clinical significance in managing liver diseases and beyond. By synthesizing existing literature, we will examine the utility of the De Ritis ratio as a diagnostic and prognostic tool in different medical contexts. Furthermore, we will discuss its limitations, potential applications beyond liver diseases, and avenues for future research.

## Review

Historical overview of the De Ritis ratio

Discovery and Development

The De Ritis ratio, also known as the AST/ALT ratio, was first described by Fernando De Ritis in 1957 and has been utilized as a liver function test to differentiate causes of liver damage or hepatotoxicity [4,5]. The ratio is calculated by dividing the AST concentration by the ALT concentration in the blood [6]. Initially, the De Ritis ratio was identified as a characteristic of acute viral hepatitis, where ALT is usually higher than AST [4,5]. However, subsequent studies have shown that the ratio is also useful in cases of alcoholic hepatitis, where AST is typically higher than ALT [4,5]. The ratio reflects the progression and severity of the disease, with an elevated AST/ALT ratio being predictive of long-term complications such as fibrosis and cirrhosis in chronic viral illnesses such as chronic viral hepatitis and chronic alcoholism [4]. The De Ritis ratio has been studied in various clinical contexts, including colorectal cancer (CRC), where a high ratio has been associated with poor overall survival (OS) and disease-free survival (DFS) in patients with stage II/III CRC [5]. Additionally, it has been linked to mortality in adult trauma patients and COVID-19, indicating its potential as a prognostic marker [4]. While the ratio does not vary significantly across different stages of fibrosis, it may need adjustment for gender. In nonalcoholic steatohepatitis (NASH), an increased AST/ALT ratio is associated with the development of cirrhosis [4]. The ratio is typically over 3.0 in newborn infants but should decrease to below 2.0 by day five; persistent elevation may indicate hepatic disease [4]. The De Ritis ratio has been used for over 60 years as a liver function test to differentiate causes of liver damage or hepatotoxicity. It represents the progression and severity of the disease, with an elevated ratio predicting long-term complications such as fibrosis and cirrhosis in chronic viral illnesses such as chronic viral hepatitis and chronic alcoholism [4]. The ratio has also been associated with mortality in adult trauma patients and COVID-19, highlighting its potential as a prognostic marker.

Evolution of Understanding

The understanding of the De Ritis ratio has evolved significantly since its initial description by Fernando De Ritis in 1957 as a simple ratio between AST and ALT levels [4]. Initially, the De Ritis ratio was primarily associated with liver diseases, such as acute viral hepatitis, serving as a marker of disease severity and progression. However, as research progressed, the significance of this ratio expanded beyond liver diseases to include other conditions such as cardiovascular disease (CVD) and COVID-19, and even as a prognostic biomarker for mortality in various patient populations [7]. Recent studies have highlighted the De Ritis ratio's association with disease severity, mortality, and prognosis in diverse clinical settings. For instance, in COVID-19 patients, an elevated De Ritis ratio has been linked to in-hospital mortality, indicating its potential as a prognostic biomarker. Moreover, the ratio's role in assessing cardiovascular risk factors, diabetes mellitus, and metabolic syndrome has been explored, showcasing its broader implications beyond liver health [8].

Previous Applications in Clinical Medicine

The De Ritis ratio has been valuable in clinical medicine for several decades, particularly in assessing liver disease. First described by Fernando De Ritis in 1957, this ratio measures the serum activities of AST and ALT [9]. It is commonly used to differentiate between causes of liver damage, with a ratio of 2:1 or greater being suggestive of alcoholic liver disease, especially in the presence of elevated gamma-glutamyl transferase [10]. Additionally, the De Ritis ratio has been identified as a predictor of fatal outcomes in COVID-19 patients, where a cutoff value of 1.218 is associated with a 2.3-fold higher risk of poor outcomes [11]. The evolution of clinical research has been a long and fascinating journey. The first recorded trial of legumes in biblical times and the first randomized controlled trial of streptomycin in 1946 highlight the progression of clinical trials over centuries [10]. The field of controlled trials has advanced significantly, incorporating ethical guidelines such as the Nuremberg Code, the Declaration of Helsinki, the Belmont Report, and the 1996 International Conference on Harmonization Good Clinical Practice guidance [10]. As governments recognized the need to address the ethical and regulatory challenges of scientific advances, clinical trials became more regulated [10]. Technological advances have continually driven clinical medicine forward, with innovations in drug design and discovery, imaging modalities, and the use of electronic medical records [12]. The De Ritis ratio exemplifies a tool developed and refined over time to improve the assessment and management of liver disease. The ongoing evolution of clinical research and technology will continue to shape the practice of clinical medicine, presenting new challenges and opportunities as scientific advancements occur.

Understanding the De Ritis ratio

Definition and Calculation

The De Ritis ratio, or AST/ALT ratio, is a biochemical metric used in clinical medicine to assess liver function and disease severity. It is calculated by dividing the serum activity of AST by the serum activity of ALT [4]. This ratio is a dynamic biochemical parameter that can change throughout a disease. For instance, in the early stages of nonalcoholic fatty liver disease (NAFLD), the De Ritis ratio may be lower due to increased ALT activity. However, at the fibrotic-cirrhotic stage, it tends to increase due to hepatic cell destruction and the release of AST from mitochondria [7]. Generally, ALT is more specific for mild liver damage, while AST is more sensitive [7]. The reference range for the De Ritis ratio is not standardized and can vary widely. Some studies suggest ideal values ranging from less than 1 to up to 1.3 in men and 1.7 in European adolescents [7]. The De Ritis ratio has been associated with various clinical conditions, including CVD, adult trauma, and COVID-19. In CVD, an elevated De Ritis ratio correlates positively with age, systemic inflammation, and impaired renal function and, inversely, with obesity, metabolic syndrome, and diabetes mellitus [7]. In adult trauma, a high De Ritis ratio has been associated with mortality and may serve as a proxy measure for ischemic end-organ damage [13]. In COVID-19, an elevated De Ritis ratio has been linked to disease severity and mortality [14]. The De Ritis ratio is a valuable biomarker in clinical medicine, providing useful information for risk evaluations and as a prognostic marker of in-hospital mortality in COVID-19 patients [13,14]. However, considering the reference range and underlying clinical condition, its interpretation should be made within the specific clinical context.

Clinical Significance

In COVID-19, a high De Ritis ratio has been associated with increased mortality [7,13]. One study found that patients with a De Ritis ratio ≥ 1.218 are more susceptible to liver damage and cytokine release syndrome [7]. Another study reported that an elevated De Ritis ratio on admission was independently associated with a more than 29-fold higher chance of in-hospital mortality in COVID-19 patients [7]. In adult trauma patients, a high De Ritis ratio has also been linked to mortality [14]. A study found that patients with a De Ritis ratio ≥ 1.6 had significantly worse survival chances and a 2.3-fold higher risk of poor outcomes [14]. In renal cell carcinoma, a high De Ritis ratio has been associated with worse survival outcomes [15]. A meta-analysis revealed that patients with an increased pretreatment De Ritis ratio had worse OS and cancer-specific survival (CSS) outcomes [15]. The De Ritis ratio is a valuable biomarker in clinical medicine, with significant applications in COVID-19, adult trauma, and renal cell carcinoma. Its utility as a prognostic biomarker in these contexts underscores the importance of monitoring this ratio in clinical practice.

Role of the De Ritis ratio in disease diagnosis

Liver Diseases

The De Ritis ratio, also known as the AST/ALT ratio, has proven to be a useful diagnostic and prognostic tool for liver diseases, including alcoholic liver disease (ALD) and chronic viral hepatitis [4,16]. In ALD, the De Ritis ratio can be as high as 2.30:1 in patients, compared to 1.10:1 in the control group, facilitating diagnosis [16]. This ratio is also helpful in distinguishing between various sources of liver disease and serves as an important predictive tool for mortality in adult trauma patients [13]. In chronic viral illnesses, such as chronic viral hepatitis and chronic alcoholism, an elevated AST/ALT ratio predicts long-term complications, including fibrosis and cirrhosis [4]. The De Ritis ratio also provides insights into the time course and aggressiveness of diseases such as acute viral hepatitis and alcoholic hepatitis [4]. However, it is important to note that methodological issues can impact the usefulness of the assays or the ratio. For instance, the presence or absence of pyridoxal phosphate in transaminase assays can affect results [4]. Using pyridoxal phosphate-supplemented assays in patients who may be pyridoxine-depleted, such as those with alcoholism, elderly individuals, and cancer patients, can help mitigate these issues. The De Ritis ratio is a valuable diagnostic and prognostic tool for liver diseases, including ALD and chronic viral hepatitis. It provides crucial disease progression and aggressiveness information and can predict long-term complications and mortality. However, careful consideration of methodological factors is essential to ensure accurate and useful assay results.

Other Medical Conditions

The De Ritis ratio, primarily used in diagnosing and managing liver diseases, has also shown utility in other medical conditions. In chronic kidney disease (CKD), a high De Ritis ratio has been associated with an increased risk of mortality [17]. Similarly, in chronic obstructive pulmonary disease (COPD), a high De Ritis ratio correlates with worse outcomes, including increased mortality and hospitalization rates [18]. Beyond liver diseases and CKD, the De Ritis ratio has been studied in various other medical contexts, such as cancer, diabetes, and CVD. For instance, in cancer patients, a high De Ritis ratio has been linked to poorer prognosis and increased mortality, particularly in liver cancer [19]. In diabetes, an elevated De Ritis ratio is associated with insulin resistance and NAFLD [20]. In CVD, a high De Ritis ratio correlates with an increased risk of cardiovascular events, such as myocardial infarction and stroke [21]. However, it is important to note that the De Ritis ratio is not a specific diagnostic tool for these conditions and should be used with other clinical assessments and tests. Additionally, the optimal cutoff value for the De Ritis ratio may vary depending on the specific medical condition and population being studied. While the De Ritis ratio is primarily used in liver diseases, it can also be useful in other medical conditions such as CKD, COPD, cancer, diabetes, and CVD. Nevertheless, it should be employed alongside other clinical assessments and tests, with consideration given to the appropriate cutoff values for each specific condition and population being studied [18].

De Ritis ratio as a prognostic indicator

Predictive Value in Disease Progression

A study demonstrated that a higher De Ritis ratio at admission was associated with increased 180-day mortality among septic patients. The De Ritis ratio was independently associated with mortality and outperformed other indicators in predicting long-term outcomes. This highlights the importance of the De Ritis ratio as a valuable prognostic tool in managing sepsis patients, providing critical information that can influence treatment decisions and patient care [22]. The De Ritis ratio was identified as an independent prognostic factor in adult patients. Higher levels of the De Ritis ratio were associated with specific characteristics and laboratory values, underscoring its potential as a prognostic indicator in this population. This suggests that the De Ritis ratio could be used to predict outcomes and guide clinical decisions, offering a significant advantage in managing adult patients across various medical conditions [23]. Research on colorectal cancer patients revealed that the De Ritis ratio could independently predict OS and DFS. Patients with a high De Ritis ratio had significantly worse OS and DFS rates, highlighting its prognostic value in this context. This indicates that the De Ritis ratio can be crucial in assessing prognosis and tailoring treatment strategies for CRC patients, ultimately improving patient outcomes [5]. A high De Ritis ratio was associated with mortality in adult trauma patients, indicating its potential as a prognostic marker in this patient population. This association suggests that the De Ritis ratio could be essential in evaluating trauma cases' severity and potential outcomes, helping clinicians make more informed decisions about patient care and intervention strategies [13].

Prognostic Significance in Survival Rates

The prognostic significance of survival rates is critical to cancer management, providing healthcare professionals with essential information to estimate prognosis and guide treatment decisions. The five-year relative survival rate is commonly used, representing the percentage of people expected to be alive five years after diagnosis, excluding those who die from unrelated causes [24]. Patient-reported outcomes (PROs) are valuable indicators in cancer patient prognostication. Studies indicate that PROs may be superior to traditional variables, such as performance status (PS), in predicting survival outcomes and are being considered for stratification in clinical trials [25]. Laboratory data also play a significant role in prognostication. Higher albumin, hemoglobin, or lymphocyte count levels are generally associated with better survival rates. In contrast, elevated alkaline phosphatase, white blood cell count, neutrophil count, and lactate dehydrogenase (LDH) often correlate with poorer survival outcomes [26]. In the context of COVID-19, the prognostic significance of the De Ritis ratio has been investigated. A De Ritis ratio ≥ 1.218 has been linked to a 2.3-fold higher risk of poor outcomes and significantly diminished chances of survival [24]. This underscores the potential of the De Ritis ratio as a valuable biomarker in predicting outcomes in COVID-19 patients, complementing other prognostic indicators in clinical practice.

Clinical utility of the De Ritis ratio

Screening Tool in Primary Care Settings

The De Ritis ratio, calculated by dividing the serum level of AST by the ALT, has demonstrated significant clinical utility as a screening tool in primary care settings. Research indicates its ability to predict a range of clinical outcomes, including shock, multiorgan failure, and mortality in conditions such as pulmonary embolism, as well as all-cause mortality in diabetic patients and elderly subjects [5,7,27]. In primary care patients, the De Ritis ratio has also been linked to the risk of CVD in men, underscoring its potential as a predictive marker in this context [7]. Furthermore, in patients with stage II/III colorectal cancer, the De Ritis ratio has been identified as an independent predictor of OS and DFS, highlighting its prognostic value in oncology settings [5]. The De Ritis ratio is a valuable parameter in primary care, providing insights into liver function and disease severity and showing promise as a screening tool for predicting various clinical outcomes across diverse patient populations. Continued research and clinical validation are crucial to fully establish the role of the De Ritis ratio in primary care settings and further refine its clinical applications.

Monitoring Disease Progression and Response to Treatment

The De Ritis ratio, also known as the AST/ALT ratio, is valuable in clinical medicine for predicting various clinical outcomes and monitoring disease progression across different contexts. In patients with advanced pancreatic ductal adenocarcinoma (PDAC) undergoing treatment with gemcitabine/nab-paclitaxel, a high pretreatment AST/ALT ratio has been associated with poorer disease outcomes and a lower response rate [28]. This highlights its potential as a prognostic indicator in cancer therapy settings. For patients with intermediate- and high-risk pulmonary embolisms, a high baseline AST/ALT ratio has been linked to outcomes such as shock, multiorgan failure, and mortality [27], indicating its utility in assessing disease severity and prognosis in acute medical conditions. The De Ritis ratio is commonly used in liver disease to monitor therapy effectiveness, disease progression, and prognosis. Elevated liver enzymes or an elevated De Ritis ratio are independently associated with increased all-cause mortality and cancer-related mortality in patients with liver diseases [7]. Moreover, in non-muscle invasive bladder cancer (NMIBC), a high pretreatment De Ritis ratio has been associated with worse recurrence-free survival (RFS) and progression-free survival (PFS) [29]. Similarly, in metastatic castration-resistant prostate cancer patients, a high De Ritis ratio correlates with poorer OS outcomes [30].

Differential Diagnosis in Ambiguous Cases

The De Ritis ratio, elevated in various diseases such as liver damage from different causes, diabetes mellitus, cancer, multiorgan failure syndromes, CVD, stroke, and COVID-19, consistently shows associations with prognosis across these conditions. This underscores its potential as a nontraditional cardiometabolic marker and a prognostic indicator in clinical practice [7]. In COVID-19, an elevated De Ritis ratio independently correlates with mortality, liver injury, and cytokine release syndrome, which are crucial in risk assessment and severity evaluation [14,31]. Its utility extends to predicting outcomes in COVID-19 patients, guiding clinical decisions, and optimizing patient management strategies [31]. The De Ritis ratio is a valuable parameter in clinical practice by aiding in the differential diagnosis of various conditions, including liver-associated diseases, CVDs, and COVID-19. Its association with disease severity, mortality, and prognosis highlights its significance as a diagnostic and prognostic marker, particularly in cases where diagnoses may be uncertain or complex.

Methodological considerations and limitations

Factors Affecting the De Ritis Ratio

**Figure 1:**
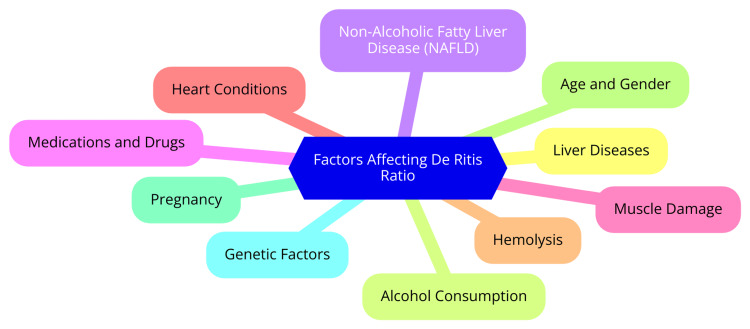
Factors affecting the De Ritis ratio Image credit: Dr Suhail Shaikh NAFLD: nonalcoholic fatty liver disease

The De Ritis ratio, also known as the AST/ALT ratio, has emerged as a significant biomarker with prognostic implications in various clinical settings. In sepsis patients, a retrospective analysis highlighted its role as a predictor of increased 180-day mortality. Patients who did not survive had notably higher De Ritis ratio values at admission compared to survivors. Multivariate Cox regression analyses confirmed the De Ritis ratio as an independent predictor of mortality, surpassing other commonly used indicators such as the sequential organ failure assessment (SOFA) score, ALT, AST, creatinine, and urea nitrogen. The receiver operating characteristic (ROC) curve analysis further underscored the superior predictive accuracy of the De Ritis ratio [22]. In trauma patients, studies have similarly identified a high De Ritis ratio (≥ 1.5) as a significant factor associated with mortality. This underscores its utility as a marker of disease severity and prognosis in critically injured individuals [13]. Additionally, among renal cell cancer patients undergoing surgery, a postoperative De Ritis ratio threshold of 1.5 was found to correlate with increased mortality risk, highlighting its prognostic value in oncological contexts [13]. In severe fever with thrombocytopenia syndrome (SFTS), the De Ritis ratio has shown correlations with multiple biomarkers indicative of disease severity, including activated partial thromboplastin time, D-dimer, fibrin degradation product, thrombin time, creatine kinase, and procalcitonin. A study established an optimal De Ritis ratio cutoff of 2.683 for predicting prognosis, demonstrating its potential as a valuable prognostic indicator in infectious disease settings [6]. Furthermore, an elevated De Ritis ratio may reflect disproportionate AST activity relative to ALT activity in patients with coronary artery disease, regardless of whether these enzymatic levels fall within or outside the standard reference range. Research has consistently linked a higher De Ritis ratio with a poorer prognosis in this patient population, underscoring its relevance in cardiovascular risk assessment [32]. Figure 1 shows the factors affecting the De Ritis ratio.

Interpretation Challenges

The De Ritis ratio has emerged as a significant marker of disease severity and mortality across various conditions, such as COVID-19, adult trauma patients, and pulmonary embolisms. However, interpreting this ratio poses several challenges [13]. Firstly, it should be evaluated alongside aminotransferase levels. A De Ritis ratio >1 alongside abnormal aminotransferase levels typically signifies liver damage, whereas a lower ratio may indicate increased ALT activity due to heightened expression or mild noxious stimuli. Disease progression can influence the De Ritis ratio, with early stages of NAFLD showing a lower ratio due to heightened ALT activity. In contrast, fibrotic-cirrhotic stages tend to elevate the ratio due to hepatic cell destruction and AST release from mitochondria [4]. Secondly, standardizing the reference range for the De Ritis ratio proves challenging. Reported values range widely, from <1 to 1.3 in men and up to 1.7 in European adolescents. This reflects the broad spectrum of AST and ALT activities in serum and complicates establishing a definitive reference range [33]. Thirdly, the relationship between the De Ritis ratio and CVD risk factors is multifaceted. Elevated De Ritis ratios correlate positively with age, systemic inflammation, and impaired renal function while showing an inverse correlation with obesity, metabolic syndrome, and diabetes mellitus. However, the exact nature and direction of this association fail to fully elucidate the link between an elevated De Ritis ratio and increased risk of CVD and cardiovascular mortality [34]. These complexities underscore the need to carefully interpret the De Ritis ratio in clinical practice, considering the context of aminotransferase levels, disease progression, and demographic factors. Ongoing research is essential to refine our understanding and utilization of this biochemical marker across various medical contexts.

## Conclusions

The comprehensive review of the De Ritis ratio underscores its pivotal role across various aspects of clinical medicine. This ratio, defined as the quotient of serum AST to ALT, is crucial for diagnosing and managing liver-related conditions while also proving useful in identifying cardiac and muscular diseases. Clinically, it enhances diagnostic accuracy, allowing for more apparent distinctions between disease types and contributing to more precise initial assessments. It is also a cost-effective, noninvasive prognostic tool that can guide patient management by monitoring disease progression or response to treatment, especially in chronic conditions. In terms of clinical practice, integrating the De Ritis ratio into routine diagnostic panels could reduce healthcare costs and improve patient outcomes through better disease management strategies. Its value in primary care and specialized settings suggests broad applicability and the potential for widespread use. Future research should focus on expanding the understanding of the De Ritis ratio through longitudinal and comparative studies across different demographics to explore variations in normal ranges and diagnostic power. Additionally, integrating this ratio into automated diagnostic algorithms could refine its utility, making it a more robust part of multimodal diagnostic approaches. Exploring the impact of genetic, lifestyle, and environmental factors on the De Ritis ratio could also contribute to advancing personalized medicine and tailoring interventions to individual patient profiles for optimal outcomes. The enduring relevance of the De Ritis ratio in clinical settings underscores its potential for enhancing patient care through both traditional and innovative medical approaches.
